# Low Intake of Vegetables and Fruits and Risk of Colorectal Cancer: The Japan Collaborative Cohort Study

**DOI:** 10.2188/jea.JE20130195

**Published:** 2014-09-05

**Authors:** Norihiro Aoyama, Miyuki Kawado, Hiroya Yamada, Shuji Hashimoto, Koji Suzuki, Kenji Wakai, Sadao Suzuki, Yoshiyuki Watanabe, Akiko Tamakoshi

**Affiliations:** 1 Department of Hygiene, Fujita Health University School of Medicine, Toyoake, Aichi, Japan; 1 藤田保健衛生大学医学部衛生学講座; 2 Department of Public Health, Fujita Health University School of Health Sciences, Toyoake, Aichi, Japan; 2 藤田保健衛生大学医療科学部公衆衛生学; 3 Department of Preventive Medicine, Nagoya University Graduate School of Medicine, Nagoya, Japan; 3 名古屋大学大学院医学研究科予防医学; 4 Department of Public Health, Nagoya City University Graduate School of Medical Sciences, Nagoya, Japan; 4 名古屋市立大学大学院医学研究科公衆衛生学分野; 5 Department of Epidemiology for Community Health and Medicine, Kyoto Prefectural University of Medicine Graduate School of Medical Science, Kyoto, Japan; 5 京都府立医科大学大学院医学研究科地域保健医療疫学; 6 Department of Public Health, Hokkaido University Graduate School of Medicine, Sapporo, Japan; 6 北海道大学大学院医学研究科公衆衛生学分野

**Keywords:** colorectal cancer, vegetables, fruits, food intake, epidemiology

## Abstract

**Background:**

The evidence for an association between low intake of vegetables and fruits and increased colorectal cancer risk is inconclusive. Evaluating the colorectal cancer risk associated with continued low intake is important.

**Methods:**

We used data of 45 516 and 14 549 subjects aged 40–79 years obtained in the baseline and interim surveys, respectively, from the Japan Collaborative Cohort Study for Evaluation of Cancer Risk (JACC Study). The intake frequency of vegetables and fruits as assessed by a self-administered questionnaire was classified into tertiles of low, middle, and high groups, and the low group was subdivided into 2 equal groups (lower low and higher low groups). Colorectal cancer incidence determined from follow-up was used. Cox’s proportional hazard model was employed to estimate the hazard ratios (HRs) and 95% confidence intervals (CIs), adjusting for covariates.

**Results:**

During 598 605 person-years of subject follow-up after baseline, we identified 806 colorectal cancer cases. HRs for the lower low versus the middle and high intake frequencies of vegetables and fruits at baseline were 0.95 (95% CI 0.77–1.16) and 1.08 (95% CI 0.90–1.29), respectively. During 125 980 person-years of subject follow-up after the interim survey, 197 colorectal cancer cases were identified. HRs for the low versus middle and high intake frequencies of vegetables and fruits in both baseline and interim surveys were 0.91 (95% CI 0.61–1.37) and 0.87 (95% CI 0.59–1.27), respectively.

**Conclusions:**

Our results suggest that low intake and continued low intake of vegetables and fruits are not strongly associated with colorectal cancer risk.

## Abstract (ja)

**【背景】:**

野菜と果物の低摂取と大腸がんの高リスクとの関連についてのエビデンスは必ずしも十分でない。低摂取の継続に対する大腸がんリスクを評価することが重要である。

**【方法】:**

基礎資料として、JACC Studyにおける40～79歳の45,516人のベースライン調査データと14,549人の中間調査データを用いた。自記式質問票による野菜と果物の摂取頻度を低・中・高摂取頻度に3区分し、低摂取頻度をさらに2つに細分した。追跡調査による大腸がん罹患の有無に基づいて、ハザード比と95％信頼区間を、Cox回帰により交絡変数を調整して推定した。

**【結果】:**

ベースライン後の598,605人年の追跡により、大腸がん罹患806人が観察された。ベースライン時の中・高頻度摂取に対する低頻度摂取（細分の低い方）における大腸がんハザード比は、野菜で0.95（95％信頼区間0.77-1.16）、果物で1.08（95％信頼区間0.90-1.29）であった。一方、中間調査後の125,980人年の追跡により、大腸がん罹患197人が観察された。ベースライン調査と中間調査ともに、中・高頻度摂取の継続に対する低頻度摂取の継続における大腸がんハザード比は、野菜で0.91（95％信頼区間0.61-1.37）、果物で0.87（95％信頼区間0.59-1.27）であった。

**【結論】:**

これらの結果から、野菜と果物の低摂取および低摂取の継続は大腸がんリスクとの強い関連がないことが示唆される。

## INTRODUCTION

The association between vegetable and fruit intake and colorectal cancer risk has been investigated by many epidemiologic studies.^1^^–^^9^ Recent cohort studies have reported no effect or a weak protective association.^5^^–^^9^ A 2007 report from the World Cancer Research Fund and the American Institute for Cancer Research concluded that there was limited suggestive evidence for reduction in colorectal cancer risk by fruits and non-starchy vegetables.^10^ The update project report in 2011 stated that recent evidence was consistent with the previous conclusion.^11^ However, those reports on vegetable and fruit intake and colorectal cancer risk were based on review of the results from cohort studies mainly in Europe and the United States, with few studies conducted in Asia (including Japan).^4^^,^^8^

Research on the association between vegetable and fruit intake and colorectal cancer risk is still inconclusive, and further studies are warranted.^11^^–^^13^ The hypothesis that low intake of vegetables and fruits could increase colorectal cancer risk has been proposed but has not been sufficiently tested.^2^^,^^6^^,^^12^ In most cohort studies, the intakes of vegetables and fruits were obtained only at baseline and were used for estimating the colorectal cancer incidence risk during the period of long-term follow-up.^12^^,^^14^ Because dietary habits could have changed during the period of follow-up, the data on food intake at baseline might not sufficiently reflect long-term exposure.^15^ We think that evaluating the colorectal cancer risk associated with continued low intake of vegetables and fruits using repeated measurements data for intake of vegetables and fruits is important. The Japan Collaborative Cohort Study for Evaluation of Cancer Risk (JACC Study) is a large-scale population-based cohort study in which the baseline and interim surveys included questionnaires assessing intake frequency of vegetables and fruits.^16^

We examined the association between vegetable and fruit intakes and the risk of colorectal cancer by analyzing the data from the baseline and interim surveys and the follow-up in the JACC Study. The focus of our analysis was to evaluate the colorectal cancer risk for participants with low intake of vegetables and fruits at baseline and continued low intake in the baseline and interim surveys.

## METHODS

### The JACC Study

The JACC Study aims to confirm the associations between cancer risk and lifestyle and living conditions.^16^ The baseline survey was conducted in 45 areas from 1988 to 1990, and 110 585 Japanese adults aged 40–79 completed a self-administered questionnaire. The interim survey was conducted in 31 areas about 5 years after the baseline survey, and 46 650 participants completed the questionnaire. The questionnaires of the baseline and interim surveys included the same questions about intake frequency of vegetables and fruits.

Of 110 585 participants at baseline, subjects for the present analysis were restricted to 65 042 participants who lived in 24 areas where information on cancer incidence was available.^16^

The study protocol was approved by the Ethical Research Committee of Nagoya University School of Medicine.

### Subjects

We analyzed colorectal cancer risk for intake frequency of vegetables and fruits at baseline using the data from the baseline survey. Out of 65 042 participants with information on cancer incidence at baseline, 3290 with a cancer history at baseline and 16 236 with no answers on intake frequency of vegetables and fruits in the baseline questionnaire were excluded. Thus, 45 516 subjects (18 946 males and 26 570 females) were left for analysis of colorectal cancer risk for intake frequency at baseline.

We performed another analysis of colorectal cancer risk associated with change in intake frequency of vegetables and fruits using the data of baseline and interim surveys. Because the interim survey was not conducted in 14 study areas, and the target of the interim survey included only some of the participants who completed the baseline survey,^15^^,^^16^ out of 45 516 subjects for analysis of intake frequency at baseline, we excluded 5576 who lived outside the study areas in the interim survey, 24 705 who had no data on intake frequency of vegetables and fruits in the interim survey, 300 subjects who had a cancer history between the baseline and interim survey, and 386 who had no information on cancer incidence after the interim survey. Thus, 14 549 subjects (5548 males and 9001 females) were left for analysis of colorectal cancer risk associated with change in intake frequency of vegetables and fruits.

### Vegetable and fruit intake and other factors

The questionnaire administered during the baseline survey covered lifestyle factors, including diet, smoking and drinking, and physical activity, as well as medical history, education, family history of cancer, height, and weight.^16^ The questionnaire administered during the interim survey included diet, smoking and drinking, medical history, and family history of cancer, but not physical activity, education, height, or weight.

We inquired about average intake frequency in the past year for 5 vegetable items (spinach, carrot and pumpkin, tomato, cabbage and lettuce, and Chinese cabbage) and 2 fruit items (orange and other fruits) in baseline and interim surveys.^17^^,^^18^ We used five response categories and assigned numerical scores to their categories: 0 for almost never, 0.5 for 1–2 times per month, 1.5 for 1–2 times per week, 3.5 for 3–4 times per week, and 6 for almost every day. Scores were averaged over items of vegetables and fruits.

We classified intake frequency of vegetables into tertile groups according to scores: <1.9 (low), 1.9–3.0 (middle), and ≥3.1 (high) for males, and <2.3 (low), 2.3–3.5 (middle), and ≥3.6 (high) for females. The group of low food frequency was subdivided into 2 equal groups: <1.3 (lower low) and 1.3–1.8 (higher low) for males, and <1.6 (lower low) and 1.6–2.2 (higher low) for females. Also, we classified intake frequency of fruits into tertile groups according to scores: <1.8 (low), 1.8–3.7 (middle), and ≥3.8 (high) for males, and <3.3 (low), 3.3–4.8 (middle), and ≥4.9 (high) for females. The group of low food frequency was subdivided into 2 equal groups: <1.0 (lower low) and 1.0–1.7 (higher low) for males, and <1.5 (lower low) and 1.5–3.2 (higher low) for females.

For the analysis of colorectal cancer risk associated with change in food frequency, we classified these food frequency groups in the baseline and interim surveys into combination groups. For example, subjects in the low food frequency group in the baseline survey and middle food frequency group in the interim survey was denoted as “low to middle”.

### Follow-up

In the JACC Study, population registries in the municipalities were used for follow-up of the subjects.^16^ In most areas, follow-up was completed at the end of 2009; however, it was stopped at the end of 1999 in 4 areas, at the end of 2003 in another 4 areas, and at the end of 2008 in 2 areas. We discontinued follow-up of those who had moved out of their area after the baseline survey.

We identified the incidence of cancer by linkage with the records of population-based cancer registries, supplemented by a systematic review of death certificates.^19^ In 4 out of 24 areas, population-based cancer registries were not available, so hospital-based cancer registries or inpatient records of hospitals treating cancer patients were used. The mortality-to-incidence ratio of cancer from the follow-up data, which served as an index for completeness of identification of cancer incidence, was reported as 0.43 in males and 0.38 in females, but the percentage of cases identified from a death certificate only (DCO%) was not.^19^

We defined colon cancer as C18 and rectum cancer as C20 according to the 10th Revision of the International Statistical Classification of Diseases and the Related Health Problems.

### Statistical analysis

For the analysis of colorectal cancer risk associated with intake frequency of vegetables and fruits at baseline, we used the baseline survey data and the follow-up data from the response of baseline survey (the registration of JACC study) in 1988–1990 to the end of the follow-up in 2009. The hazard ratios (HRs) and 95% confidence intervals (CIs) for colorectal cancer according to intake frequency of vegetables and fruits at baseline (the HRs for lower low, and higher low food frequencies versus the middle and high food frequencies) were estimated using Cox’s proportional hazard models. For the analysis of colorectal cancer risk for change in intake frequency of vegetables and fruits, we used the data from baseline and interim surveys and the follow-up data from the responses to the interim survey to the end of the follow-up in 2009. The HRs and 95% CIs for colorectal cancers according to change in intake frequency of vegetables and fruits (the HRs for low to low, low to middle and high, and middle and high to low food frequencies versus middle and high to middle and high food frequency) were estimated.

For the analysis of colorectal cancer risk for intake frequency of vegetables and fruits at baseline, we adjusted for the following potential confounding factors at baseline: sex, age (40–44 years, 45–49, 50–54, 55–59, 60–64, 65–69, 70–74, or 75–79), district (Hokkaido and Tohoku, Kanto, Tokai, Kinki, Chugoku, or Kyushu), smoking (current smoker of 20 or more cigarettes per day, current smoker of 1–19 cigarettes per day or unknown numbers, former smoker, or nonsmoker), drinking (current drinker with everyday drinking, current drinker without everyday drinking, former drinker, or nondrinker), intake frequency of beef and pork (tertile groups according to scores: <2.0, 2.0–3.4, or ≥3.5 times per week), family history of colorectal cancer in parents and/or siblings (yes or no), education (attended school until ≥18 years, or <18 years), body mass index calculated as weight (kg)/height (m)^2^ (<18.5, 18.5–24.9, or ≥25.0), and walking time (≥30 min/day or <30). For the analysis of colorectal cancer risk for change in intake frequency of vegetables and fruits, we adjusted for sex, age, district, smoking, drinking, intake frequency of beef and pork, and family history of colorectal cancer in parents and/or siblings at the interim survey, and education, body mass index, and walking time at baseline. Missing values for each covariate were treated as an additional category in the variables and were included in the Cox’s proportional hazard model. All analyses were performed using the SAS statistical package, version 9.3 (SAS, Institute, Cary, NC, USA).

## RESULTS

Table 1 shows the characteristics of subjects for analysis of colorectal cancer risk associated with intake frequency of vegetables and fruits at baseline. The average age at baseline was 56.7 years. The proportions of lower low intake frequency of vegetables and fruits at baseline were 15.9% and 19.1%, respectively. The proportion of current smokers was 52.7% in males and 5.1% in females. The proportion of body mass index of ≥25 kg/m^2^ was 20.8%.

**Table 1.** Characteristics of subjects for analysis of colorectal cancer incidence for food frequency at baseline

Characteristics at baseline	Males	Females	All
Number of subjects	18 946	26 570	45 516
Age (years)			
mean, standard deviation	56.5, 10.2	56.9, 10.0	56.7, 10.1
Distinct (%)			
Hokkaido & Tohoku	8.5	8.9	8.8
Kanto	22.2	34.3	29.3
Chubu	9.5	9.8	9.7
Kinki	49.5	38.9	43.3
Chugoku & Kyushu	10.3	8.1	9.1
Intake frequency^a^			
Vegetables (%)			
Lower low	15.4	16.2	15.9
Higher low	17.2	15.2	16.0
Middle	31.9	36.1	34.4
High	35.4	32.5	33.7
Fruits (%)			
Lower low	21.5	17.4	19.1
Higher low	11.9	14.7	13.5
Middle	30.2	38.8	35.2
High	36.4	29.2	32.2
Other characteristics			
Smoking (%)			
Current smoker of 20 or more cigarettes per day	34.9	1.4	15.7
Current smoker of 1–19 cigarettes per day or unknown numbers	17.8	3.7	9.7
Former smoker	26.3	1.5	12.1
Nonsmoker	20.9	93.4	62.5
Drinking (%)			
Current drinker with everyday drinking	52.4	5.5	25.1
Current drinker without everyday drinking	23.3	18.4	20.5
Former drinker	6.2	1.8	3.6
Nondrinker	18.1	74.4	50.9
Intake frequency of beef and pork (%)^a^			
<2.0 times per week	37.4	36.3	36.8
2.0–3.4 times per week	25.4	27.0	26.3
≥3.5 times per week	37.2	36.7	36.9
Family history of colorectal cancer (%)			
Yes	2.4	2.8	2.6
No	97.6	97.2	97.4
Education (%)			
Attended school until			
≥18 years	20.7	11.3	15.2
<18 years	79.3	88.7	84.8
Body mass index (%)			
<18.5 kg/m^2^	5.1	6.1	5.7
18.5–24.9 kg/m^2^	76.1	71.7	73.5
≥25.0 kg/m^2^	18.9	22.1	20.8
Walking time (%)			
≥30 min/day	68.5	71.7	70.3
<30 min/day	31.5	28.3	29.7

^a^Intake frequency of vegetables was grouped according to scores: <1.3 (lower low), 1.3–1.8 (higher low), 1.9–3.0 (middle) and ≥3.1 (high) for males, and <1.6 (lower low), 1.6–2.2 (higher low), 2.3–3.5 (middle) and ≥3.6 (high) for females. Intake frequency of fruits was grouped: <1.0 (lower low), 1.0–1.7 (higher low), 1.8–3.7 (middle) and ≥3.8 (high) for males, and <1.5 (lower low), 1.5–3.2 (higher low), 3.3–4.8 (middle) and ≥4.9 (high) for females. Intake frequency scores were averaged over items in vegetables, fruits, and beef and pork.

Table 2 shows the characteristics of subjects for analysis of colorectal cancer risk associated with change in intake frequency of vegetables and fruits. The average age at the interim survey was 60.8 years. The proportions of low to low intake frequency of vegetables and fruits were 16.8% and 21.9%, respectively. The proportion of current smokers was 43.4% in males and 3.6% in females. The proportion of body mass index of 25 kg/m^2^ and over was 18.8%.

**Table 2.** Characteristics of subjects for analysis of colorectal cancer incidence for change in food frequency

Characteristics at interim survey	Males	Females	All
Number of subjects	5548	9001	14 549
Age (years)			
mean, standard deviation	61.1, 9.4	60.6, 9.3	60.8, 9.3
Distinct (%)			
Hokkaido & Tohoku	2.0	1.9	1.9
Kanto	40.5	51.3	47.2
Chubu	5.9	4.0	4.7
Kinki	39.1	33.5	35.6
Chugoku & Kyushu	12.6	9.3	10.5
Intake frequency^a^			
Vegetables (%)			
Low to low	17.6	16.3	16.8
Low to middle	9.6	10.9	10.4
Low to high	4.6	3.7	4.0
Middle to low	9.6	9.8	9.8
Middle to middle	13.8	18.2	16.5
Middle to high	10.0	9.7	9.8
High to low	5.6	3.6	4.4
High to middle	11.1	11.1	11.1
High to high	18.0	16.7	17.2
Fruits (%)			
Low to low	22.5	21.5	21.9
Low to middle	9.7	8.4	8.9
Low to high	3.5	1.8	2.5
Middle to low	11.8	13.8	13.1
Middle to middle	12.0	19.1	16.4
Middle to high	7.1	6.4	6.7
High to low	5.9	4.6	5.1
High to middle	10.1	12.8	11.8
High to high	17.4	11.5	13.8
Other characteristics			
Smoking (%)			
Current smoker of 20 or more cigarettes per day	28.3	1.0	11.7
Current smoker of 1–19 cigarettes per day or unknown numbers	15.1	2.5	7.5
Former smoker	31.3	1.4	13.2
Never smoked	25.2	95.1	67.6
Drinking (%)			
Current drinker with everyday drinking	55.6	6.5	25.6
Current drinker without everyday drinking	19.5	16.3	17.6
Former drinker	4.4	1.3	2.5
Never drinked	20.5	75.9	54.3
Intake frequency of beef and pork (%)^a^			
<2.0 times per week	34.3	31.0	32.3
2.0–3.4 times per week	17.8	20.4	19.4
≥3.5 times per week	47.8	48.6	48.3
Family history of colorectal cancer (%)			
Yes	7.4	7.6	7.5
No	92.6	92.4	92.5
Education (%)^b^			
Attended school until			
≥18 years	22.4	11.8	15.8
<18 years	77.6	88.2	84.2
Body mass index (%)^b^			
<18.5 kg/m^2^	4.7	5.8	5.4
18.5–24.9 kg/m^2^	78.1	74.5	75.9
≥25.0 kg/m^2^	17.2	19.7	18.8
Walking time (%)^b^			
≥30 min/day	71.4	73.8	72.9
<30 min/day	28.6	26.2	27.1

^a^Intake frequency of vegetables was grouped according to scores: <1.9 (low), 1.9–3.0 (middle) and ≥3.1 (high) for males, and <2.3 (low), 2.3–3.5 (middle) and ≥3.6 (high) for females. Intake frequency of fruits was grouped: <1.8 (low), 1.8–3.7 (middle) and ≥3.8 (high) for males, and <3.3 (lower low), 3.3–4.8 (middle) and ≥4.9 (high) for females. Intake frequency scores were averaged over items of vegetables, fruits, and beef and pork. These food frequency groups in the baseline and interim surveys were classified into combination groups. For example, participants in the low food frequency group in the baseline survey and middle food frequency group in the interim survey were denoted as “low to middle”.

^b^Data were from the baseline survey.

During 598 605 person-years of subject follow-up for analysis of colorectal cancer risk associated with intake frequency of vegetables and fruits at baseline, there were 552 cases of colon cancer (299 males and 253 females) and 254 cases of rectal cancer (168 males and 86 females). The DCO% in colorectal cancer cases was 8.4%. The mortality-to-incidence ratio was 0.35. During 125 980 person-years of subject follow-up for analysis of colorectal cancer risk associated with change in intake frequency of vegetables and fruits, there were 137 cases of colon cancer (76 males and 61 females) and 60 cases of rectal cancer (43 males and 17 females). The DCO% in colorectal cancer cases was 6.6%. The mortality-to-incidence ratio was 0.40.

Table 3 shows the HR and 95% CI for colorectal cancer incidence for analysis of intake frequency of vegetables and fruits at baseline, adjusted for sex, age, district, smoking, drinking, intake frequency of beef and pork, family history of colorectal cancer, education, body mass index, and walking time. We found no significant association between the low intake frequency of vegetables or fruits and colorectal cancer incidence. HR of colorectal cancer incidence for the lower low versus the middle and high intake frequencies of vegetables and fruits at baseline was 0.95 (95% CI 0.77–1.16) and 1.08 (95% CI 0.90–1.29), respectively. HRs of colon or rectal cancer incidence for the lower low versus the middle and high intake frequencies of vegetables or fruits at baseline in males or females ranged from 0.59 to 1.32.

**Table 3.** Hazard ratio (HR) and 95% confidence interval (CI) for colorectal cancer incidence for analysis of food frequency at baseline

Intake frequency at baseline		Males				Females				All			
		
		Number of cases	HR^a^ (95% CI)		*P* value	Number of cases	HR^a^ (95% CI)		*P* value	Number of cases	HR^a^ (95% CI)		*P* value
For colon cancer incidence													
Vegetables:	Lower low	41	0.98	(0.70–1.38)	0.904	45	1.17	(0.84–1.63)	0.356	86	1.07	(0.85–1.36)	0.559
	Higher low	55	1.10	(0.81–1.48)	0.553	29	0.82	(0.56–1.22)	0.334	84	1.00	(0.79–1.26)	0.975
	Middle & high	203	1.00			179	1.00			382	1.00		

Fruits:	Lower low	70	1.12	(0.84–1.48)	0.451	40	0.90	(0.63–1.28)	0.567	110	1.04	(0.83–1.29)	0.741
	Higher low	28	0.83	(0.55–1.24)	0.353	38	1.05	(0.73–1.50)	0.800	66	0.93	(0.72–1.22)	0.603
	Middle & high	201	1.00			175	1.00			376	1.00		

For rectal cancer incidence													
Vegetables:	Lower low	15	0.59	(0.34–1.01)	0.054	13	0.93	(0.51–1.71)	0.823	28	0.69	(0.46–1.03)	0.070
	Higher low	26	0.85	(0.56–1.30)	0.460	10	0.78	(0.40–1.53)	0.477	36	0.84	(0.59–1.20)	0.342
	Middle & high	127	1.00			63	1.00			190	1.00		

Fruits:	Lower low	41	1.32	(0.91–1.92)	0.150	12	0.90	(0.48–1.69)	0.739	53	1.17	(0.85–1.61)	0.331
	Higher low	18	1.01	(0.61–1.68)	0.971	14	1.27	(0.70–2.30)	0.425	32	1.06	(0.72–1.55)	0.772
	Middle & high	109	1.00			60	1.00			169	1.00		

For colorectal cancer incidence													
Vegetables:	Lower low	56	0.83	(0.62–1.11)	0.208	58	1.11	(0.83–1.48)	0.490	114	0.95	(0.77–1.16)	0.592
	Higher low	81	1.00	(0.78–1.28)	0.988	39	0.81	(0.58–1.14)	0.233	120	0.95	(0.78–1.15)	0.578
	Middle & high	330	1.00			242	1.00			572	1.00		

Fruits:	Lower low	111	1.18	(0.94–1.48)	0.145	52	0.90	(0.66–1.22)	0.495	163	1.08	(0.90–1.29)	0.412
	Higher low	46	0.89	(0.65–1.22)	0.463	52	1.10	(0.81–1.49)	0.553	98	0.97	(0.78–1.21)	0.789
	Middle & high	310	1.00			235	1.00			545	1.00		

^a^Hazard ratio was adjusted for sex, age, district, smoking, drinking, intake frequency of beef and pork, family history of colorectal cancer, education, body mass index, and walking time at baseline.

Table 4 shows the HRs and 95% CIs for colorectal cancer incidence for analysis of change in intake frequency of vegetables and fruits, adjusted for sex, age, district, smoking, drinking, intake frequency of beef and pork, family history of colorectal cancer, education, body mass index, and walking time. We found no significant association between the low to low intake frequency of vegetables or fruits and colorectal cancer incidence. HRs of colorectal cancer incidence for the low to low versus middle and high to middle and high intake frequency of vegetables and fruits were 0.91 (95% CI 0.61–1.37) and 0.87 (95% CI 0.59–1.27), respectively. HRs of colon or rectal cancer incidence for the low to low versus middle and high to middle and high intake frequency of vegetables or fruits in males or females ranged from 0.57 to 2.03.

**Table 4.** Hazard ratio (HR) and 95% confidence interval (CI) for colorectal cancer incidence for analysis of change in food frequency

Change in intake frequency		Males				Females				All			
		
		Number of cases	HR^a^ (95% CI)		*P* value	Number of cases	HR^a^ (95% CI)		*P* value	Number of cases	HR^a^ (95% CI)		*P* value
For colon cancer incidence													
Vegetables:	Low to low	16	1.21	(0.67–2.20)	0.533	6	0.59	(0.25–1.42)	0.241	22	0.92	(0.57–1.50)	0.749
	Low to middle & high	10	0.88	(0.44–1.77)	0.720	5	0.48	(0.19–1.21)	0.118	15	0.68	(0.39–1.19)	0.174
	Middle & high to low	10	0.88	(0.44–1.78)	0.727	8	0.88	(0.41–1.90)	0.753	18	0.86	(0.51–1.44)	0.564
	Middle & high to middle & high	40	1.00			42	1.00			82	1.00		

Fruits:	Low to low	17	0.96	(0.51–1.82)	0.901	15	1.17	(0.60–2.28)	0.644	32	1.03	(0.65–1.63)	0.892
	Low to middle & high	9	0.93	(0.44–1.98)	0.851	4	0.65	(0.23–1.88)	0.429	13	0.80	(0.44–1.48)	0.481
	Middle & high to low	19	1.47	(0.81–2.66)	0.204	14	1.27	(0.66–2.46)	0.474	33	1.37	(0.89–2.13)	0.155
	Middle & high to middle & high	31	1.00			28	1.00			59	1.00		

For rectal cancer incidence													
Vegetables:	Low to low	5	0.58	(0.22–1.55)	0.274	4	2.03	(0.61–6.81)	0.252	9	0.88	(0.41–1.87)	0.741
	Low to middle & high	6	0.77	(0.31–1.89)	0.566	2	0.90	(0.19–4.17)	0.887	8	0.81	(0.37–1.76)	0.592
	Middle & high to low	6	0.73	(0.30–1.80)	0.497	1	0.60	(0.08–4.80)	0.630	7	0.70	(0.31–1.59)	0.394
	Middle & high to middle & high	26	1.00			10	1.00			36	1.00		

Fruits:	Low to low	9	0.58	(0.26–1.33)	0.198	2	0.57	(0.12–2.77)	0.488	11	0.57	(0.28–1.17)	0.127
	Low to middle & high	3	0.35	(0.11–1.18)	0.091	1	0.39	(0.05–3.06)	0.367	4	0.37	(0.13–1.05)	0.063
	Middle & high to low	6	0.52	(0.21–1.31)	0.165	4	1.29	(0.39–4.33)	0.676	10	0.67	(0.33–1.38)	0.279
	Middle & high to middle & high	25	1.00			10	1.00			35	1.00		

For colorectal cancer incidence													
Vegetables:	Low to low	21	0.96	(0.58–1.60)	0.885	10	0.83	(0.42–1.67)	0.607	31	0.91	(0.61–1.37)	0.650
	Low to middle & high	16	0.84	(0.48–1.46)	0.531	7	0.56	(0.25–1.23)	0.146	23	0.73	(0.46–1.14)	0.163
	Middle & high to low	16	0.83	(0.48–1.44)	0.507	9	0.83	(0.41–1.69)	0.602	25	0.81	(0.53–1.26)	0.355
	Middle & high to middle & high	66	1.00			52	1.00			118	1.00		

Fruits:	Low to low	26	0.80	(0.49–1.32)	0.388	17	1.02	(0.56–1.87)	0.939	43	0.87	(0.59–1.27)	0.468
	Low to middle & high	12	0.66	(0.35–1.25)	0.203	5	0.58	(0.23–1.48)	0.252	17	0.64	(0.38–1.07)	0.090
	Middle & high to low	25	1.04	(0.64–1.70)	0.862	18	1.25	(0.70–2.23)	0.446	43	1.12	(0.77–1.62)	0.554
	Middle & high to middle & high	56	1.00			38	1.00			94	1.00		

^a^Hazard ratio was adjusted for sex, age, district, smoking, drinking, intake frequency of beef and pork and family history of colorectal cancer at the interim survey, and education, body mass index, and walking time at baseline.

## DISCUSSION

Analysis of data from a population-based cohort study in Japan (the JACC Study) showed no significant associations between the intake frequency of vegetables or fruits and colorectal cancer incidence. Many recent cohort studies in Europe and the United States have found no or a weak protective association between vegetable and fruit intakes and colorectal cancer incidence.^5^^,^^6^^,^^9^^,^^11^ Our results are consistent with those of other cohort studies for Japanese inhabitants, showing no substantial protective associations.^4^^,^^20^

A focus of our analysis was to evaluate the colorectal cancer risk for having low intake of vegetables and fruits. We used the lower low intake frequency group (less than 20% of our subjects) as the low food intake category in our analysis. HRs for the lower low intake frequencies of vegetables and fruits at baseline was not significant at 0.95 and 1.08, respectively. It has been hypothesized that low vegetable and fruit consumption beyond a certain level leads to an increase in colorectal cancer risk,^6^^,^^12^^,^^13^ although the level is not clearly defined.^12^ Although the lower low intake frequency of vegetables and fruits in the present study might not be below the level described in the hypothesis, our results do not support the hypothesis.

Another focus of our analysis was to evaluate the colorectal cancer risk associated with continued low intake of vegetables and fruits. In most cohort studies on colorectal cancer risk, vegetable and fruit intakes have been measured only at baseline.^12^^,^^14^ Such data might lead to underestimation of the colorectal cancer risk associated with low vegetable and fruit intakes during the period of follow-up, because diets may change considerably over time.^12^^,^^15^ Although we found no significant increased risk of colorectal cancer in the low to low intake frequency group, it should be noted that our results were obtained through analysis partly considering the change in intake frequency of vegetables and fruits over time and therefore provide useful information for evaluating the colorectal cancer risk associated with vegetable and fruit intake.

Some problems and limitations in the present study warrant mention. We used the intake frequency of vegetables and fruits assessed by a self-administered questionnaire but did not obtain the actual vegetable and fruit intake. HRs of low intake of vegetables and fruits for colorectal cancer risk based on data of intake frequency in the present study may therefore not be entirely accurate. However, the questionnaire of food frequencies has been validated by referring to four 3-day weighed dietary records over a 1-year period as a standard and was found to be suitable for use in a large group of subjects.^18^ Most members of the low, middle, and high intake frequency groups in our analysis would be low, middle, and high in intake, respectively. Thus, based on the amounts of intake, HRs of low intake of vegetables and fruits for colorectal cancer risk should not substantially differ from our estimates.

For identification of cancer incidence in the JACC Study, population-based cancer registries were available in 20 areas, but hospital-based cancer registries or inpatient records of hospitals treating cancer patients were instead used in 4 areas.^19^ The completeness of identification for cancer incidence varies across study areas. In our two analyses, the DCO% (8.4% and 6.6%) and mortality-to-incidence ratio (0.35 and 0.40) for colorectal cancer were comparable to those from representative population-based cancer registries in Japan.^21^

The analysis of colorectal cancer risk associated with intake frequency of vegetables and fruits at baseline based on 806 cases of colorectal cancer seemed to have considerable statistical power of significance.^10^ In contrast, only 197 cases of colorectal cancer were available for the analysis of changes in intake frequency of vegetables and fruits. Thus, the HRs for low to low frequency of vegetable and fruit intake would include considerable uncertainty. Further studies with sufficient statistical power to confirm these findings are therefore necessary. We adjusted for some covariates in the Cox’s proportional hazard models, and although the covariates were similar to those from previous reports on the association between vegetable and fruit intake and colorectal cancer risk, possible residual confounding cannot be ruled out.^4^^,^^20^

In conclusion, our results suggested that low intake and continued low intake of vegetables and fruits were not strongly associated with colorectal cancer risk.

## ONLINE ONLY MATERIAL

Abstract in Japanese.

## References

[1] Terry P, Giovannucci E, Michels KB, Bergkvist L, Hansen H, Holmberg L, Fruit, vegetables, dietary fiber, and risk of colorectal cancer. J Natl Cancer Inst. 2001;93:525–33 10.1093/jnci/93.7.525 11287446

[2] McCullough ML, Robertson AS, Chao A, Jacobs EJ, Stampfer MJ, Jacobs DR, A prospective study of whole grains, fruits, vegetables and colon cancer risk. Cancer Causes Control. 2003;14:959–70 10.1023/B:CACO.0000007983.16045.a1 14750535

[3] Wark PA, Weijenberg MP, van ’t Veer P, van Wijhe G, Lüchtenborg M, van Muijen GN, Fruits, vegetables, and hMLH1 protein-deficient and -proficient colon cancer: The Netherlands cohort study. Cancer Epidemiol Biomarkers Prev. 2005;14:1619–25 10.1158/1055-9965.EPI-05-0109 16030092

[4] Sato Y, Tsubono Y, Nakaya N, Ogawa K, Kurashima K, Kuriyama S, Fruit and vegetable consumption and risk of colorectal cancer in Japan: The Miyagi Cohort Study. Public Health Nutr. 2005;8:309–14 10.1079/PHN2004681 15918928

[5] McCarl M, Harnack L, Limburg PJ, Anderson KE, Folsom AR Incidence of colorectal cancer in relation to glycemic index and load in a cohort of women. Cancer Epidemiol Biomarkers Prev. 2006;15:892–6 10.1158/1055-9965.EPI-05-0700 16702366

[6] Park Y, Subar AF, Kipnis V, Thompson FE, Mouw T, Hollenbeck A, Fruit and vegetable intakes and risk of colorectal cancer in the NIH-AARP diet and health study. Am J Epidemiol. 2007;166:170–80 10.1093/aje/kwm067 17485731

[7] Nomura AM, Wilkens LR, Murphy SP, Hankin JH, Henderson BE, Pike MC, Association of vegetable, fruit, and grain intakes with colorectal cancer: the Multiethnic Cohort Study. Am J Clin Nutr. 2008;88:730–7 18779290 10.1093/ajcn/88.3.730 PMC4482464

[8] Lee SA, Shu XO, Yang G, Li H, Gao YT, Zheng W Animal origin foods and colorectal cancer risk: a report from the Shanghai Women’s Health Study. Nutr Cancer. 2009;61:194–205 10.1080/01635580802419780 19235035 PMC2810117

[9] van Duijnhoven FJ, Bueno-De-Mesquita HB, Ferrari P, Jenab M, Boshuizen HC, Ros MM, Fruit, vegetables, and colorectal cancer risk: the European Prospective Investigation into Cancer and Nutrition. Am J Clin Nutr. 2009;89:1441–52 10.3945/ajcn.2008.27120 19339391

[10] World Cancer Research Fund/American Institute of Cancer Research. Food, Nutrition, Physical Activity, and the Prevention of Cancer: a Global Perspective. Washington DC: American Institute of Cancer Research; 2007.

[11] World Cancer Research Fund/American Institute for Cancer Research. Continuous Update Project Report. Food, Nutrition, Physical Activity, and the Prevention of Colorectal Cancer. 2011.

[12] Aune D, Lau R, Chan DS, Vieira R, Greenwood DC, Kampman E, Nonlinear reduction in risk for colorectal cancer by fruit and vegetable intake based on meta-analysis of prospective studies. Gastroenterology. 2011;141:106–18 10.1053/j.gastro.2011.04.013 21600207

[13] Johnson CM, Wei C, Ensor JE, Smolenski DJ, Amos CI, Levin B, Meta-analyses of colorectal cancer risk factors. Cancer Causes Control. 2013;24:1207–22 10.1007/s10552-013-0201-5 23563998 PMC4161278

[14] Koushik A, Hunter DJ, Spiegelman D, Beeson WL, van den Brandt PA, Buring JE, Fruits, vegetables, and colon cancer risk in a pooled analysis of 14 cohort studies. J Natl Cancer Inst. 2007;99:1471–83 10.1093/jnci/djm155 17895473

[15] Suzuki S, Kawado M, Hashimoto S, Tokudome S, Yoshimura T, Tamakoshi A; JACC Study Group Change in food intake frequency at five years after baseline in the JACC study. J Epidemiol. 2005;15 Suppl 1:S48–55 10.2188/jea.15.S48 15881195 PMC8565870

[16] Tamakoshi A, Ozasa K, Fujino Y, Suzuki K, Sakata K, Mori M, Cohort profile of the Japan Collaborative Cohort Study at final follow-up. J Epidemiol. 2013;23:227–32 10.2188/jea.JE20120161 23583921 PMC3700254

[17] Iso H, Date C, Noda H, Yoshimura T, Tamakoshi A; JACC Study Group Frequency of food intake and estimated nutrient intake among men and women: the JACC Study. J Epidemiol. 2005;15 Suppl 1:S24–42 10.2188/jea.15.S24 15881193 PMC8565867

[18] Date C, Fukui M, Yamamoto A, Wakai K, Ozeki A, Motohashi Y, Reproducibility and validity of a self-administered food frequency questionnaire used in the JACC study. J Epidemiol. 2005;15 Suppl 1:S9–23 10.2188/jea.15.S9 15881192 PMC8565864

[19] Mori M, Sakauchi F, Washio M, Ozasa K, Watanabe Y, Yoshimura T, Survey for incidence of cancer as a measure of outcome in the JACC study. J Epidemiol. 2005;15 Suppl 1:S80–5 10.2188/jea.15.S80 15881199 PMC8565871

[20] Tsubono Y, Otani T, Kobayashi M, Yamamoto S, Sobue T, Tsugane S; JPHC Study Group No association between fruit or vegetable consumption and the risk of colorectal cancer in Japan. Br J Cancer. 2005;92:1782–4 10.1038/sj.bjc.6602566 15856039 PMC2362020

[21] Curado MP, Edwards B, Shin HR, Storm H, Ferlay J, Heanue M, et al, editors. Cancer Incidence in Five Continents Vol. IX, Lyon: International Agency for Research on Cancer; 2007.

