# Supplementary material for: Low Intake of Vegetables and Fruits and Risk of Colorectal Cancer: The Japan Collaborative Cohort Study
Source: J Epidemiol. 2014 Sep 5;24(5):353–60. doi: 10.2188/jea.JE20130195 (PMC4150005; doi:10.2188/jea.JE20130195)
Supplement: Abstract in Japanese. [file je-24-353-s001.pdf]

野菜と果物の低摂取と大腸がんリスク：JACC Study

青山典裕[1]、川戸美由紀[1]、山田宏哉[1]、橋本修二[1]、鈴木康司[2]、若井建志[3]、鈴木貞夫[4]、渡邊能行[5]、玉腰暁子[6]、JACC Study Group

[1]藤田保健衛生大学医学部衛生学講座、[2]藤田保健衛生大学医療科学部公衆衛生学、[3]名古屋大学大学院医学研究科予防医学、[4]名古屋市立大学大学院医学研究科公衆衛生学分野、[5]京都府立医科大学大学院医学研究科地域保健医療疫学、[6]北海道大学大学院医学研究科公衆衛生学分野

【背景】野菜と果物の低摂取と大腸がんの高リスクとの関連についてのエビデンスは必ずしも十分でない。低摂取の継続に対する大腸がんリスクを評価することが重要である。

【方法】基礎資料として、JACC Study における 40～79 歳の 45,516 人のベースライン調査データと 14,549 人の中間調査データを用いた。自記式質問票による野菜と果物の摂取頻度を低・中・高摂取頻度に 3 区分し、低摂取頻度をさらに 2 つに細分した。追跡調査による大腸がん罹患の有無に基づいて、ハザード比と 95％信頼区間を、Cox 回帰により交絡変数を調整して推定した。

【結果】ベースライン後の 598,605 人年の追跡により、大腸がん罹患 806 人が観察された。ベースライン時の中・高頻度摂取に対する低頻度摂取（細分の低い方）における大腸がんハザード比は、野菜で 0.95（95％信頼区間 0.77-1.16）、果物で 1.08（95％信頼区間 0.90-1.29）であった。一方、中間調査後の 125,980 人年の追跡により、大腸がん罹患 197 人が観察された。ベースライン調査と中間調査ともに、中・高頻度摂取の継続に対する低頻度摂取の継続における大腸がんハザード比は、野菜で 0.91（95％信頼区間 0.61-1.37）、果物で 0.87（95％信頼区間 0.59-1.27）であった。

【結論】これらの結果から、野菜と果物の低摂取および低摂取の継続は大腸がんリスクとの強い関連がないことが示唆される。

キーワード：大腸がん、野菜、果物、食物摂取、疫学
